# Should Primigravidas or Secundigravidas be Routinely Screened for Cervical Insufficiency? Insights from Two Cases of Emergency Cerclage Using a Modified Macdonald Technique

**Published:** 2026-04-18

**Authors:** George Uchenna Eleje, Isaiah Chukwuebuka Umeoranefo, Chichi Ukoha, Ekeuda Uchenna Nwankwo, Emmanuel Chukwubuikem Egwuatu, Odigonma Zinobia Ikpeze, Charles Chukwuka Ezema, Obinna Kenneth Nnabuchi, Chigozie Geoffrey Okafor, Kingsley Chidiebere Nwaogu, Michel Chiedu Egbuniwe, Emeka Philip Igbodike, Kenechukwu Ezekwesili Obi, Adanna Vivian Egwim, Chukwuemeka Chukwubuikem Okoro, Chukwudubem Chinagorom Onyejiaka, Chiemezie Mac-Kingsley Agbanu, Donald Ugochukwu Nwasike, Emeka Godswill Igboekwe, Chijioke Ogomegbunam Ezeigwe, Stanley Chigaemezu Egbogu, Casmir Chukwudi Madubuko, Alexander Arinze Mathias, Martin Chinedu Andeh, Nkejesus Chidube Obi, Ebube BelieveGod Akosa, Lambert Chukwu Onyejiaku, James Egwuatu Okonkwo, Ifeanyi Kingsley Nwaeju, Tochukwu Chukwuemeka Obiechina, Chidebe Christian Anikwe, John Chukwuemeka Nkesi, Felix Nwabunwanne Emodi, Samuel Nnaji Ugadu, Ijeoma Rita Chukwuka, Florence Olumma Ndu, Emmanuel Onyebuchi Ugwu, Joseph Ifeanyichukwu Ikechebelu, Gerald Okanandu Udigwe, Ahizechukwu Chigoziem Eke

**Affiliations:** 1 Department of Obstetrics and Gynaecology, Effective Care Research Unit, Nnamdi Azikwe University, Nigeria; 2 Department of Obstetrics and Gynaecology, Nnamdi Azikwe University Teaching Hospital, Nigeria; 3 Department of Obstetrics and Gynaecology, Barts Health NHS Foundation Trust, UK; 4 Rural Community Clinical School, School of Medicine, Deakin University, Australia; 5 Department of Radiology, Nnamdi Azikwe University Teaching Hospital, Nigeria; 6 Department of Nursing, University of Sunderland, Sunderland, United Kingdom; 7 Department of Obstetrics and Gynaecology, University of Lagos, Nigeria; 8 Department of Obstetrics and Gynaecology, College of Medicine, University of Nigeria, Nigeria; 9 Division of Maternal Fetal Medicine, Department of Gynecology and Obstetrics, Johns Hopkins University School of Medicine, USA

**Keywords:** Cervical insufficiency, Emergency cerclage, Primigravida, Secondigravida, Modified MacDonald technique, Preterm Birth prevention, Cervical incompetence

## Abstract

Cervical insufficiency is a significant contributor to mid-trimester pregnancy loss and preterm birth. Emergency cervical cerclage is a potentially life-saving intervention, but its use remains controversial, especially in primigravidas or secundigravidas with no identifiable risk factors. We report two cases involving a primigravida and a secundigravida, both presenting with painless cervical dilation and bulging fetal membranes at 22- and 21-weeks’ gestation, respectively. Neither had a history of cervical procedures or prior pregnancy losses. Following emergency transvaginal cervical cerclage the first case progressed to a live birth at 36 weeks gestation while the second, unfortunately experienced pregnancy loss at 23 weeks. We presented these case reports to highlight that cervical insufficiency could present unexpectedly in women without traditional risk factors, and that emergency cerclage may improve outcomes, but risks remain. Routine second-trimester cervical length screening may therefore be warranted in primigravidas or secundigravidas to identify at-risk pregnancies and allow for timely elective intervention.

## Introduction

Cervical insufficiency is a clinical condition characterized by painless cervical dilation in the second trimester in the absence of uterine contractions, resulting in pregnancy loss or extreme prematurity [1,2]. It is implicated in approximately 0.5% to 1% of all pregnancies and accounts for 15% to 25% of second-trimester losses [3]. Despite its relatively low prevalence, the burden of cervical insufficiency is significant, particularly due to its association with spontaneous preterm birth, which remains a leading cause of neonatal morbidity and mortality worldwide [4,5].

Emergency cervical cerclage, performed after cervical dilation and membrane bulging has already occurred, is a key intervention for cervical insufficiency. While prophylactic and ultrasound-indicated cerclage procedures are more established [4], the efficacy of emergency cerclage remains contentious. Some studies suggest it can prolong pregnancy and improve neonatal outcomes even in advanced cervical changes [6,7], while others caution against its use due to increased risks of infection, Preterm Premature Rupture of Membranes (PPROM), and procedure failure [8,9]. The lack of consensus is particularly evident in primigravidas or secundigravidas, who lack the obstetric history (e.g., previous preterm birth) that typically guides the indication for cerclage. Current guidelines, such as those from the American College of Obstetricians and Gynaecologists (ACOG), do not universally recommend routine cervical length screening in low-risk primigravidas, despite emerging evidence suggesting that some may benefit from surveillance and early intervention [10,11]. This may result in missed opportunities for early intervention, particularly in women who develop cervical insufficiency without traditional risk factors.

Surgical technique plays a pivotal role in the success of cervical cerclage procedures. While the McDonald technique remains the standard approach due to its simplicity and efficacy, various modifications have been introduced to address specific clinical challenges, particularly in cases with advanced cervical changes [4]. One such innovation is the “Ikechebelu technique,” a modified transvaginal cerclage method developed in Nigeria. This technique is characterized by enhanced suture placement aimed at achieving superior cervical reinforcement [5]. Although the Ikechebelu technique has demonstrated effectiveness in elective cerclage procedures, its application in emergency settings remains underexplored in the literature.

This report presents two cases of cervical insufficiency: one in a 24-year-old primigravida and another in a 30-year-old secundigravida, both managed with emergency cervical cerclage using the modified Ikechebelu technique in the first case and modified McDonald technique in the second case. These cases provide valuable insights into the potential role of emergency cerclage in women without traditional risk factors for cervical insufficiency. They also contribute to the limited body of evidence on the use of the Ikechebelu technique in acute settings. Furthermore, these cases reveal the need to reconsider cervical surveillance strategies, even among low-risk, first-time or second-time pregnancies.

## Case Presentation

### Case 1

#### Patient Profile

Mrs. CC is a 24-year-old Nigerian primigravida with Secondary School Education (SSCE) and occupation as a seamstress. She booked for antenatal care at 8 weeks’ gestation, following a spontaneous conception detected by urine test and confirmed with early ultrasound. Her Last Menstrual Period (LMP) was March 5, 2024, corresponding to an estimated due date of December 12, 2024.

#### Initial Presentation

At 22 weeks and 1 day gestation, she presented with a five-day history of mild, painless, bright red vaginal spotting with minimal clots. There were no associated symptoms such as abdominal pain, dizziness, fever, urinary or vaginal infections. She had no prior cervical procedures or obstetric interventions. At presentation, the bleeding had spontaneously resolved.

#### Investigations and Findings

**Figure 1:**
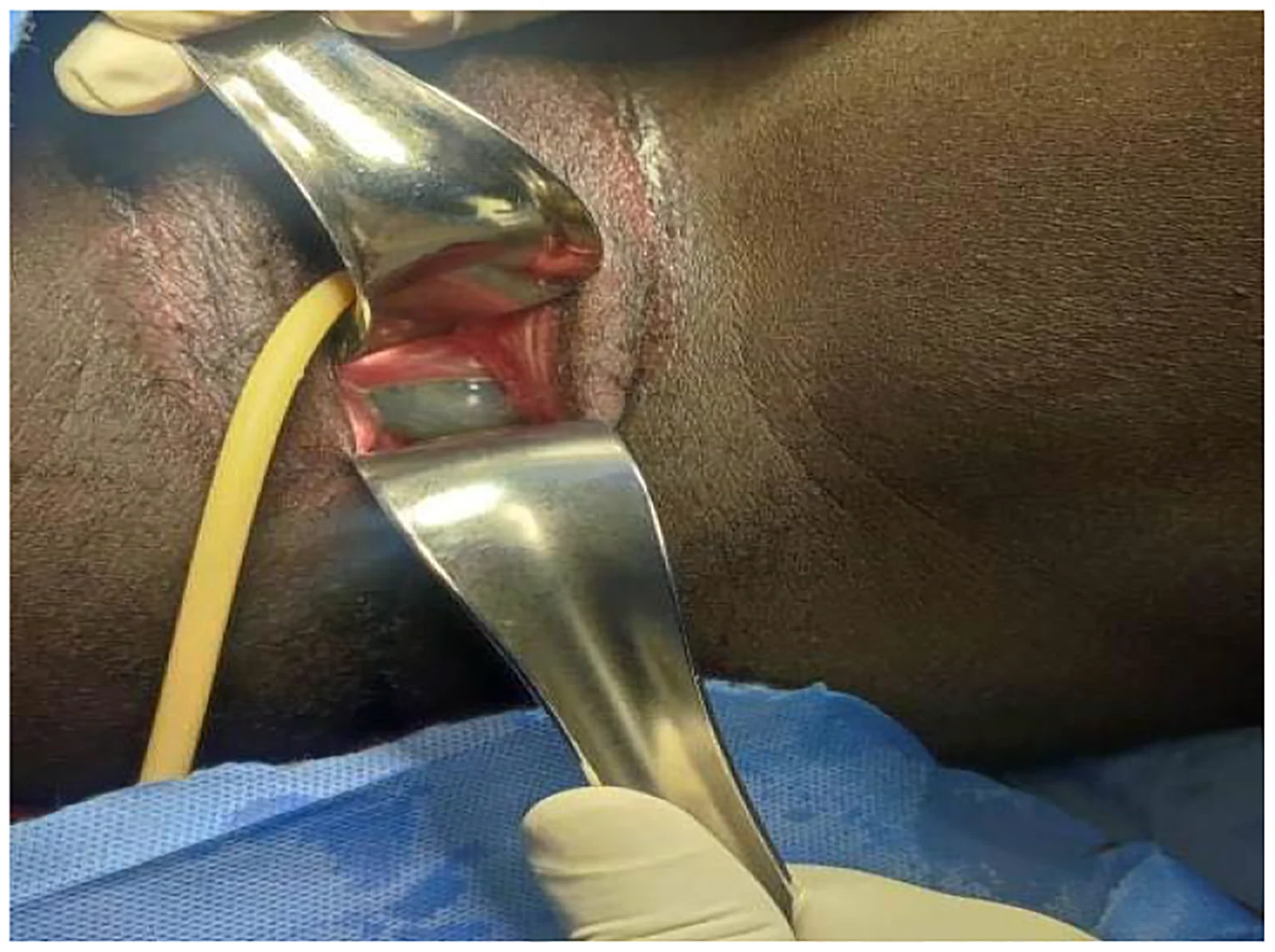
Preoperative image of the cervix.

She was advised to undergo an obstetric ultrasound, which revealed features suggestive of cervical insufficiency. The placenta was fundally situated. Subsequent speculum examination confirmed the diagnosis, revealing bulging fetal membranes protruding through a 6cm dilated cervix (Figure 1).

#### Diagnosis and Initial Management

A diagnosis of cervical insufficiency was made. She was counselled and booked for emergency transvaginal cerclage. Preoperative laboratory investigations revealed a blood group of AB Rhesus D positive, a Packed Cell Volume (PCV) of 34%, and negative results for Retroviral Screening (RVS), Hepatitis B Surface Antigen (HBsAg), and Hepatitis C Virus (HCV).

#### Surgical Intervention

**Figure 2:**
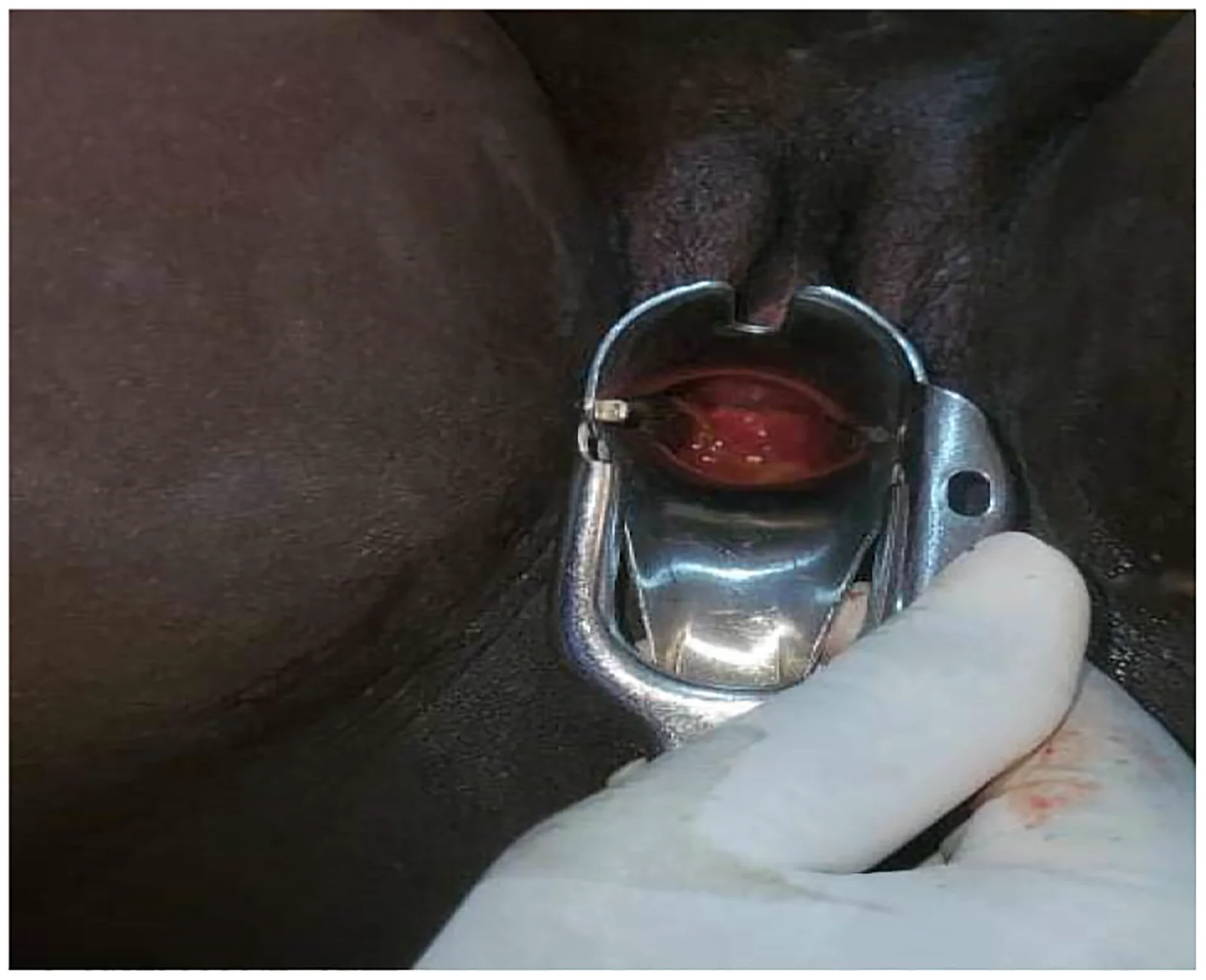
Postoperative image of the cervix on the 3^rd^ day of discharge.

Under general anaesthesia, an emergency transvaginal cerclage was performed using a modified Macdonald technique (Ikechebelu’s modification [5]). With the patient in Trendelenburg position, bulging membranes were repositioned into the uterus using a size 18 French Foley catheter balloon. Three bite triangular deep suturing with a 5mm mersilene tape was placed around the ectocervix as high as possible and tied at 6 O’clock position using a 2:1:1 surgeon’s knot configuration. The procedure was uneventful, estimated blood loss was 50 mL, and post-operative cervical os was closed (Figure 2).

#### Post-Operative Care

Postoperatively, the patient received rectal diclofenac and intramuscular pentazocine for analgesia. Tocolysis was maintained with oral nifedipine. She was administered intravenous ceftriaxone and metronidazole for antibiotic coverage, along with oral Duphaston (dydrogesterone) for hormonal support. Additional supportive treatments included oral Vasoprin, haematinics, and intravenous fluids. The patient made an uneventful recovery with no immediate postoperative complications.

#### Complication and Subsequent Management

At 27 weeks gestation, she was admitted with recurrent mild vaginal bleeding. CTG monitoring was initiated. An ultrasound scan revealed a normally positioned posterior fundal placenta. A diagnosis of threatened miscarriage with prior cervical cerclage was made. Despite conservative management (antibiotics, tocolytics, haematinics, and corticosteroids), bleeding persisted, necessitating cerclage removal. While on admission, the bleeding resolved spontaneously, and she was discharged after two weeks of hospital admission with a closed cervical os and no bulging membranes. She received two doses of dexamethasone 12-hourly. Subsequently, she was seen at 2-weekly visits, without any fresh complaints.

#### Labour and Delivery

At 36 weeks + 4 days, she presented in spontaneous active labour (5 cm cervical dilation). On examination, fetal lie was longitudinal, presentation cephalic, with no complications. Routine investigations (urinalysis, RVS, HBsAg, HCV, genotype, blood group, PCV, ultrasound, VDRL) were within normal limits. Labour monitoring revealed poor progression. Clinical pelvimetry indicated contracted mid pelvis with prominent ischial spines. An emergency caesarean section was indicated.

#### Intraoperative Care

Intraoperatively, the uterus, fallopian tubes, and ovaries were all found to be normal, with no evidence of uterine rupture or placental abnormalities. A live male infant weighing 2.55 kilograms was delivered, with APGAR scores of 6 at one minute and 9 at five minutes. The placenta, weighing approximately 0.5 kilograms, was delivered using controlled cord traction. Estimated blood loss during the procedure was 500 millilitres. The new born was admitted to the Special Care Baby Unit (SCBU) for management of neonatal jaundice and was subsequently discharged in stable condition after 48 hours.

#### Postoperative Recovery

The patient received standard postoperative care (IV fluids, antibiotics, analgesics, early ambulation). The catheter was removed after 48 hours. She remained stable and was discharged on postoperative day 4 with prescriptions for wound care and medications (ferrous sulfate, folic acid, vitamin C, metronidazole, and co-amoxiclav). Follow-up two weeks later was uneventful.

### Case 2

Mrs. XX is a 30-year-old Nigerian woman, gravida 2 para 0+1 (G2P0+1), who presented at 21 weeks and 4 days’ gestation, dated from her last normal menstrual period (October 26, 2024). She reported light vaginal spotting that began four days prior, noticed only after urination and not accompanied by clots, heavy bleeding, abdominal pain, dizziness, fever, or urinary symptoms. She had otherwise been in good health.

The current pregnancy was spontaneous and desired. Her obstetric history includes a left salpingectomy for a ruptured ectopic pregnancy at 10 weeks in June 2024. She has no history of miscarriage, cervical surgery, or uterine instrumentation.

She had booked for antenatal care and had normal initial investigations with no complications until this episode. A pelvic ultrasound revealed findings consistent with cervical insufficiency.

She was counselled and scheduled for an emergency (rescue) transvaginal cerclage. Pre-operative investigations revealed a Packed Cell Volume (PCV) of 34%, and she tested negative for HIV, hepatitis B, and hepatitis C. Her blood group was B Rhesus D positive.

#### Examination Findings

**Figure 3:**
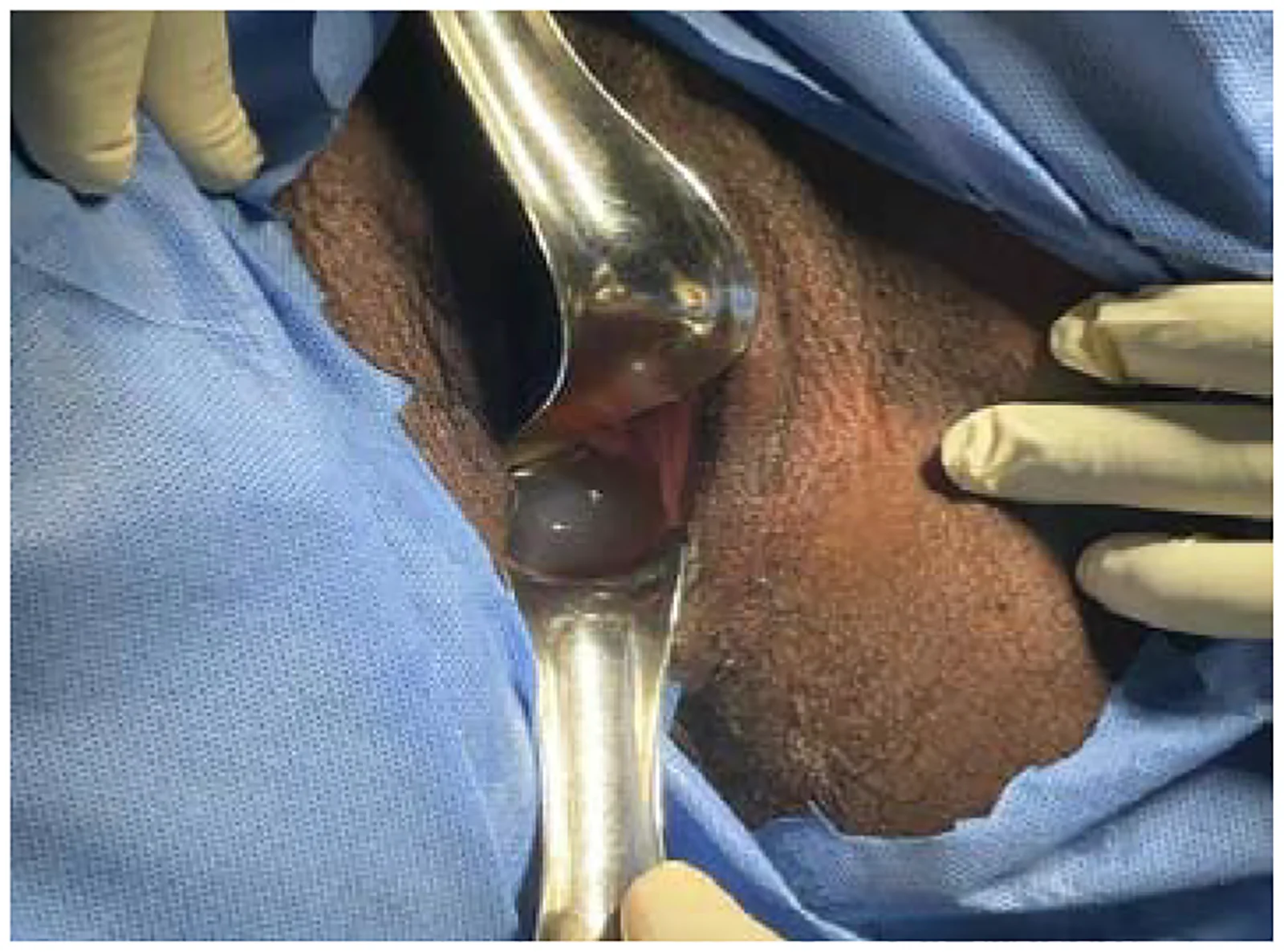
Preoperative image of the cervix.

She appeared clinically stable and well. She was afebrile (36.6°C), not pale, jaundiced, or cyanosed. No pedal oedema or dehydration was noted. Vital signs were within normal limits: BP 111/63 mmHg, pulse 78 bpm, respiratory rate 22 cycles/min, and oxygen saturation 98% on room air. Speculum examination confirmed a 6cm dilated cervix with visible bulging, unruptured fetal membranes (Figure 3). A diagnosis of cervical insufficiency in a secundigravida was made.

#### Surgical Procedure

Under spinal anaesthesia, an emergency transvaginal cerclage was performed. The patient was positioned in lithotomy, followed by the Trendelenburg position. After sterile preparation and bladder emptying, vaginal retraction was achieved with Sim’s specula. The cervix was grasped using sponge-holding forceps.

A size 18G Foley catheter was inserted into the cervical canal and inflated with 20 mL sterile water to reduce the bulging membranes. A modified McDonald cerclage technique was performed using Mersilene tape on two curved needles [1, 2]. Sutures were placed circumferentially: one needle from 11 to between 8 and 7 o’clock, and the second from from 1 to between 4 and 5 o’clock. The ends were tied at 6 o’clock, leaving 4 cm of tape. The Foley catheter was then deflated and removed. Haemostasis was secured and instruments withdrawn. The area was cleaned and the patient returned to a supine position.

Estimated blood loss was approximately 50 mL. The cervix remained closed post-procedure, and the patient’s immediate postoperative condition was stable.

#### Postoperative Care

She was commenced on intravenous Ceftriaxone at a dose of 1 gram daily for 48 hours, followed by oral Cefpodoxime 200 milligrams taken twice daily for seven days. In addition, she was placed on oral Dydrogesterone 10 milligrams three times daily for two weeks to support the pregnancy. For analgesia and inflammation control, rectal Diclofenac 100 milligrams was administered twice daily for 48 hours.

She was advised on pelvic rest and signs requiring urgent review. A size 18G Foley catheter was retained for 24 hours post-surgery. She was monitored and discharged on postoperative day 2 in satisfactory condition.

#### Outcome

**Figure 4:**
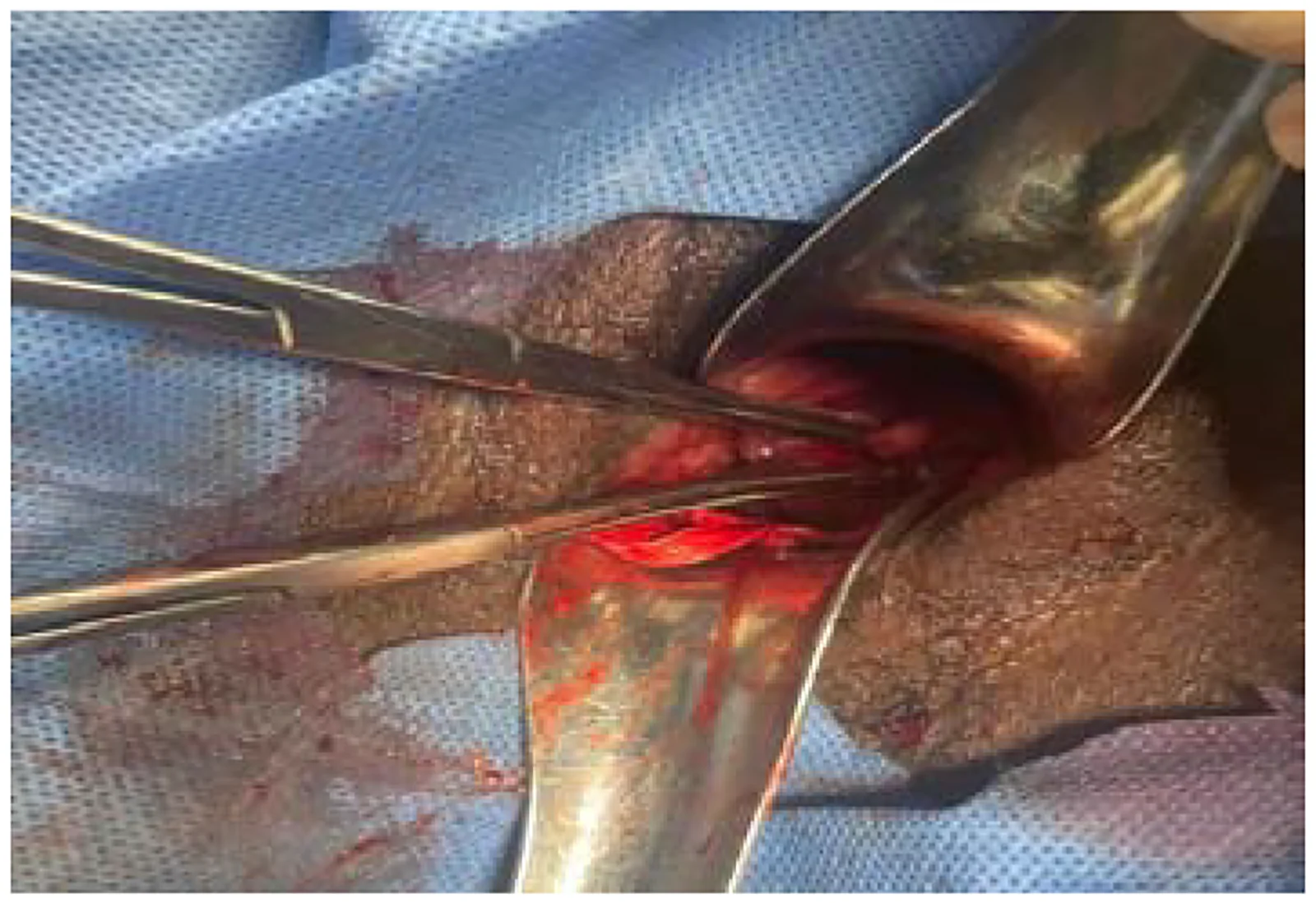
Immediate postoperative image of the cervix.

Unfortunately, despite successful cerclage placement, she experienced spontaneous rupture of membranes 14 days later, resulting in removal of the cerclage and subsequent explusion of the fetus and placenta (Figure 4).

## Discussion

These cases reveal several critical aspects of managing cervical insufficiency in a primigravida or secundigravidas with no prior risk factors. The patient’s presentation at 22 weeks’ gestation with painless cervical dilation and bulging fetal membranes required immediate intervention. The successful placement of an emergency cerclage prolonged the pregnancy to a viable gestational age in case 1, demonstrating the potential effectiveness of cervical cerclage in preventing pregnancy loss in high-risk scenarios. A recent meta-analysis supports this finding, indicating that emergency cerclage in women with painless cervical dilation can reduce preterm birth, prolong gestation, and decrease both fetal loss and neonatal mortality [7]. However, despite favourable outcomes, the evidence supporting emergency cerclage remains of low to very low quality due to the scarcity of randomised controlled trials [7].

The modified Ikechebelu technique employed in the first case was associated with minimal complications and no immediate postoperative adverse events [5]. The original Ikechebelu technique involves placing triangular “three-bite” sutures tied at the 12 o’clock position, leaving a 3 cm suture tail, based on the 3:3:3 principle [5]. In our first case, a modified approach was used (Eleje cerclage technique), securing the knot at the 6 o’clock position, which was based on the lead author’s experience. Tying the knot at 12 o’clock is often linked with bladder irritation, and the modification aimed to mitigate this risk. Despite recurrence of vaginal bleeding at 27 weeks necessitating cerclage removal in case 1, the pregnancy progressed to near term, yielding a favourable neonatal outcome.

These cases raise important questions about how to identify risk factors for cervical insufficiency in primigravidas or secundigravidas without a prior obstetric or gynaecologic history. Our patients exhibited none of the established risk factors for cervical insufficiency, suggesting that cervical insufficiency can develop even in women without identifiable predisposing conditions. This study advocates for the consideration of routine mid-trimester cervical length screening via transvaginal ultrasound during antenatal care for all pregnant women, including those in their first pregnancy.

Several factors influence the success of rescue cerclage. One major challenge lies in identifying subclinical Intra-Amniotic Infection (IAI), which cannot be diagnosed reliably through clinical evaluation alone [12]. Studies have shown that the degree of cervical dilation alone does not reliably predict perinatal outcomes [8]. Subclinical IAI, present in 13% to 50% of pregnancies requiring rescue cerclage, often remains undetected unless amniotic fluid is assessed through advanced diagnostic methods [8]. Recent evidence supports performing amniocentesis prior to cerclage placement to evaluate for immunologic, biochemical, and microbiologic markers in the amniotic fluid, which can guide decision-making and potentially improve neonatal outcomes [12]. Performing cerclage in the presence of subclinical infection has been linked with poor outcomes, a possibility in the second case given that the patient was unbooked and lacked early screening.

The successful outcome of these emergency cerclages was also facilitated by specific intraoperative techniques. The Trendelenburg position was employed to reduce pressure on the fetal membranes. Preparations included bladder emptying and continuous drainage using a Foley catheter. The cervix was visualised using Sim’s specula. An 18 French Foley catheter balloon inflated with 20 mL of sterile water was used to gently reposition bulging membranes and was removed following suture placement without causing membrane rupture. Similar methods, including using a catheter bulb or uniconcave balloon and lowering the head end of the bed, have been reported to reduce the risk of membrane puncture during cerclage [13,14].

Instruments such as vulsellum and sponge-holding forceps were used to stabilize the cervix, with care taken to avoid rupturing membranes. Prior literature describes the use of curved forceps to encircle the cervix gently, providing upward compression of the membranes and a secure field for suture placement, particularly relevant in the Shirodkar technique after bladder base dissection [15].

Our findings reinforce the importance of early detection of cervical insufficiency, particularly in primigravidas or secundigravidas. The successful use of emergency cerclage in this case is consistent with findings by Yi et al., who demonstrated that emergency cerclage between 24–28 weeks in singleton pregnancies can reduce neonatal morbidity, prolong gestation, and lower rates of preterm birth before 28, 32, and 34 weeks, as well as neonatal intensive care admissions [16].

This case report raises an important clinical question: Should routine cervical assessment be implemented in all primigravidas or secundigravidas? Evidence suggests that early identification of cervical insufficiency through cervical length screening allows for timely elective cerclage, and reduces the risk of emergency interventions. A systematic review has shown that cerclage significantly reduces preterm birth in women with cervical insufficiency, even in those without traditional risk factors [18]. For primigravidas, who may show no clinical signs until advanced cervical changes occur, routine ultrasound measurement of cervical length (particularly <25 mm) during the second trimester may serve as a critical tool in risk stratification and early management [17]. Early detection also facilitates enhanced surveillance through serial ultrasounds and physical examination.

Recent strategies to optimise emergency cerclage outcomes include performing amniocentesis with therapeutic amnioreduction prior to the procedure [8,14]. For instance, Werlang, et al demonstrated that measuring interleukin-6 (IL-6) in amniotic fluid can help identify appropriate candidates for cerclage, given IL-6’s predictive value for both infection and poor outcomes [12]. They advocate for further research into refining management protocols for high-risk patients.

### Clinical Implications

These cases highlight the importance of routine mid-trimester cervical length measurement in primigravidas and secundigravidas, even in the absence of known risk factors. Such an approach may facilitate the early detection of cervical insufficiency and enable timely intervention before significant cervical changes occur. Furthermore, the positive outcome following emergency cerclage reveals its vital role in preventing pregnancy loss in cases of advanced cervical insufficiency.

Comprehensive patient counselling is also essential to ensure that women are fully informed about the potential risks and benefits of cerclage, empowering them to make well-informed decisions regarding their care. Attention to technical details during emergency cerclage placement, including optimal patient positioning, careful membrane repositioning, and precise suture placement, can significantly influence procedural success [3,4]. The modified Ikechebelu technique used in our first case offers a promising alternative that warrants further evaluation through larger studies [5].

These case reports describe two instructive instances of cervical insufficiency in a primigravida and a secundigravida without conventional risk factors, both of whom presented in the second trimester with painless cervical dilation and bulging membranes. One case resulted in a successful live birth following emergency cerclage, while the other unfortunately ended in pregnancy loss. These contrasting outcomes reveal the clinical dilemma faced by obstetricians when managing unexpected cervical insufficiency in women with no prior obstetric or gynaecologic history suggestive of risk. We argue that routine second-trimester cervical length screening, even in low-risk gravidas, may offer opportunities for timely intervention and improved pregnancy outcomes.

### Research Implications

There is a pressing need to develop reliable predictive models for cervical insufficiency in nulliparous women. Such models would support early identification of at-risk individuals and facilitate timely preventive interventions. In addition, comparative studies are warranted to evaluate the efficacy and safety of various cervical cerclage techniques, including the modified Ikechebelu technique, in order to determine the most appropriate approaches for different clinical scenarios. These studies should consider both immediate procedural success and long-term maternal and neonatal outcomes.

Moreover, further research is essential to assess the long-term effects of emergency cerclage, as current data remain limited. This includes evaluating outcomes in subsequent pregnancies to determine the risk of recurrence of cervical insufficiency. Such evidence would be instrumental in guiding clinical decision-making and enhancing the quality of patient counselling.

## Limitations and Strengths

These case reports present several limitations that must be considered when interpreting our findings. As a case report involving only two patients, the generalizability is inherently limited, and the study does not allow for conclusions about causality or treatment efficacy. Additionally, neither patient had baseline cervical length measurements prior to the onset of advanced cervical changes, which precluded an assessment of the timing and progression of cervical shortening. This missing data limits our understanding of the natural history of cervical insufficiency in primigravidas and secundigravidas. Furthermore, the absence of long-term follow-up, particularly regarding subsequent pregnancies, restricts our ability to assess recurrence risk and the potential need for prophylactic interventions in future gestations.

Despite these limitations, the report has remarkable strengths that warrant acknowledgment. It provides detailed documentation of clinical presentation, management, and outcomes in a clinical context that remains underrepresented in the literature, specifically, cervical insufficiency in women without traditional risk factors. The successful prolongation of pregnancy in Case 1, from 22 weeks to near term, demonstrates the potential utility of emergency cerclage in primigravidas. Moreover, the use of a modified cerclage technique (the Ikechebelu modification) contributes to the limited evidence on innovative surgical approaches in emergency settings. This technique may offer a viable alternative in challenging cases with advanced cervical changes, particularly in resource-limited settings, and warrants further investigation.

Perhaps most importantly, these cases reveal a critical clinical dilemma regarding the screening of cervical insufficiency in primigravidas and secundigravidas. They raise the possibility that routine cervical length assessment in early mid-trimester antenatal care could help identify at-risk women who might otherwise go undetected until advanced cervical changes occur, thereby informing future clinical practice guidelines. While the American College of Obstetricians and Gynaecologists (ACOG) does recommend cervical length screening, our report emphasizes the potential benefit of extending this screening to low-risk primigravidas and secundigravidas, an area not yet widely implemented in low-resource settings [19]. The ACOG primarily recommends cervical length screening for women with established risk factors or a history of preterm birth [19]. Although cervical length assessment is performed during routine mid-pregnancy ultrasounds, its application in low-risk women is not universally practiced [19]. Our report reinforces the potential value of extending such screening to low-risk populations, as this could facilitate earlier identification of at-risk women and potentially prevent preterm births.

## Conclusion

These cases illustrate that cervical insufficiency can occur unexpectedly in primigravidas or secundigravidas, even in the absence of identifiable risk factors, and remains a significant cause of mid-trimester loss and preterm birth. Emergency cerclage can be a lifesaving intervention, significantly improving neonatal outcomes. Routine cervical length screening, particularly in the early mid-trimester, should be considered part of standard prenatal care in primigravidas or secundigravidas to facilitate early detection and elective intervention. The favourable outcome achieved through a modified transvaginal cerclage approach in this case highlights the potential of such techniques in improving pregnancy outcomes in resource-constrained settings. This case advocates for greater vigilance in antenatal care and supports further research into refining risk assessment and cerclage techniques for the management of cervical insufficiency.
